# Relationship between rectus abdominis muscle thickness and metabolic syndrome in middle-aged men

**DOI:** 10.1371/journal.pone.0185040

**Published:** 2017-09-15

**Authors:** Eun Sil Choi, Soo Hyun Cho, Jung-Ha Kim

**Affiliations:** Department of Family Medicine, College of Medicine, Chung-Ang University Hospital, Seoul, Korea; Waseda University, JAPAN

## Abstract

**Background and objective:**

Skeletal muscle has been suggested as an important factor in the pathophysiology of metabolic syndrome. During the aging process, muscle mass is lost in specific body parts. However, few studies have investigated the relationship between site-specific muscle loss assessed using computed tomography (CT) and metabolic syndrome. This study was conducted to investigate the association between metabolic syndrome and rectus abdominis muscle thickness at the umbilicus level (RAM), which reflects site-specific muscle loss of the abdomen using CT image.

**Methods:**

This cross-sectional study was conducted on 725 middle-aged Korean men. Anthropometric evaluation and biochemical tests were performed. The RAMs of the subjects were measured from CT images taken at the umbilicus level.

**Results:**

The mean RAM (mean ±SD) of subjects with metabolic syndrome was 2.46 ±0.01, which was thinner than that of subjects without metabolic syndrome (2.52 ±0.01, *p*<0.01). Moreover, RAM decreased as the number of metabolic syndrome components increased (p-value for trend<0.01). RAM was positively correlated with body mass index (r = 0.21, *p*<0.01), skeletal muscle index (r = 0.26, *p*<0.01), and creatinine (r = 0.12, *p*<0.01), while RAM was negatively correlated with age(r = -0.11, *p*<0.01), abdominal circumference(r = -0.22, *p*<0.01), fasting glucose (r = -0.10, *p*<0.01), and triglycerides(r = -0.15, *p*<0.01). Using a stepwise multiple logistic regression analysis, we found that RAM was an independent factor associated with metabolic syndrome (OR: 0.861, 95%CI, 0.779–0.951, *p*<0.01). The result was not different in the statistical analysis including the components of MS (OR: 0.860, 95% CI, 0.767–0.965, *p* = 0.01).

**Conclusion:**

RAM was associated with metabolic syndrome in middle-aged men. Moreover, site-specific muscle loss at the abdomen, as evaluated by RAM, also may be a predictor of metabolic syndrome like SMI.

## Introduction

The incidence of metabolic syndrome (MS), defined as a combination of metabolic abnormalities such as abdominal obesity, hyperglycemia, hypertension and dyslipidemia, is increasing in many countries worldwide, including Korea [1]. While the pathophysiology of MS is complex and incompletely understood, insulin resistance and chronic inflammation are thought to be involved [2].

Muscle contraction stimulates glucose transport to the skeletal muscles [3] and muscle strength is inversely correlated with MS [4]. Resistance exercise has been shown to be associated with decreased cardiovascular risk by mechanisms such as increased fat-free body mass, increased glycemic control, improved lipid profile, and reduced blood pressure [5]. Muscle also secretes various factors related with insulin resistance, glucose uptake, and fat oxidation in muscle; moreover, regulation of muscle and fat mass have been reported to be connected [6, 7]. Therefore, skeletal muscle has been proposed as an important factor in the pathophysiology of MS and may even be a predisposing factor for MS. While many studies have examined the relationship between muscle mass and MS, apparently conflicting results have been obtained [4, 8, 9].

Dual energy X-ray absorptiometry (DXA), magnetic resonance imaging (MRI), and computed tomography (CT) are used as measuring muscle mass [10]. Of these techniques, CT and MRI are the most accurate methods of measuring muscle mass, but their use is limited due to high cost and radiation does [10]. Therefore, DXA, which is easy to test and has low radiation dose, is generally used for clinical muscle mass measurement [10]. Muscle mass can be expressed by the skeletal muscle index (SMI), which is calculated by dividing the appendicular skeletal muscle mass (ASM) DXA estimate by the total body weight or height squared [10].

However, recent studies have reported that muscle mass loss associated with aging varies depending on the body part and have also demonstrated that most aging-related skeletal muscle loss occurs in the quadriceps and abdominal region [11, 12]. One study demonstrated a relationship between thigh muscle development and MS [13], another study showed that the risk score for MS was independently associated with rectus abdominis muscle thickness as measured by ultrasound [14]. While there is a growing body of evidence to support site-specific muscle loss, few studies have investigated the relationship between abdominal muscle and MS. Moreover, as far as we know, no studies have been done to measure the size of abdominal muscles using CT or MRI, the gold standard method of measuring muscle mass, and to demonstrate its association with the MS.

Thus, the purpose of this study was to evaluate the relationship between the rectus abdominis muscle measured using CT images and MS. We tested the hypothesis that rectus abdominis muscle thickness at the umbilicus level (RAM) assessed using CT images, which reflects rectus abdominis muscle size, is related to MS in middle-aged men.

## Methods

### Subjects

This study was conducted on 798 men aged 40–64 years who underwent health screening at the Health Promotion Center of the Chung-ang University Hospital between March 2013 and August 2016. The study included men whose health screens included abdomen computed tomography (abdomen CT) scans or abdomen-pelvis computed tomography (APCT) scans. We measured the height, weight, blood pressure (BP), and waist circumference (WC) of each subject. In addition, bioelectrical impedance analysis (BIA) and blood tests were performed. Current medication information was also obtained from patient medical records. Of the 798 subjects, 73 non-Koreans were excluded from the study, leaving 725 subjects eligible for the analysis. Each subject was assigned to either the “with MS” or “without MS” group. MS was defined as satisfying three or more of the following conditions, according to the Modified NCEP ATP Ⅲ definition[15] and the Korean Society for the Study of Obesity criteria[16]: 1) Elevated WC: WC ≥90cm in Korean men; 2) Elevated TG: Triglyceride ≥150mg/dl; 3) Reduced high-density lipoprotein (HDL) cholesterol: HDL cholesterol less than <40mg/dl; 4) Elevated BP: Systolic BP ≥130mmHg or diastolic BP ≥85mmHg or taking anti-hypertensive medication; and 5) Elevated fasting glucose: Fasting blood glucose ≥100mg/dl or taking a hypoglycemic agent or insulin therapy. The study was approved by the Institutional Review Board of Chung-Ang University Hospital. Informed written consent was obtained from all participants (IRB No: 1702-002-16037).

### Rectus abdominis muscle thickness

**Fig 1:**
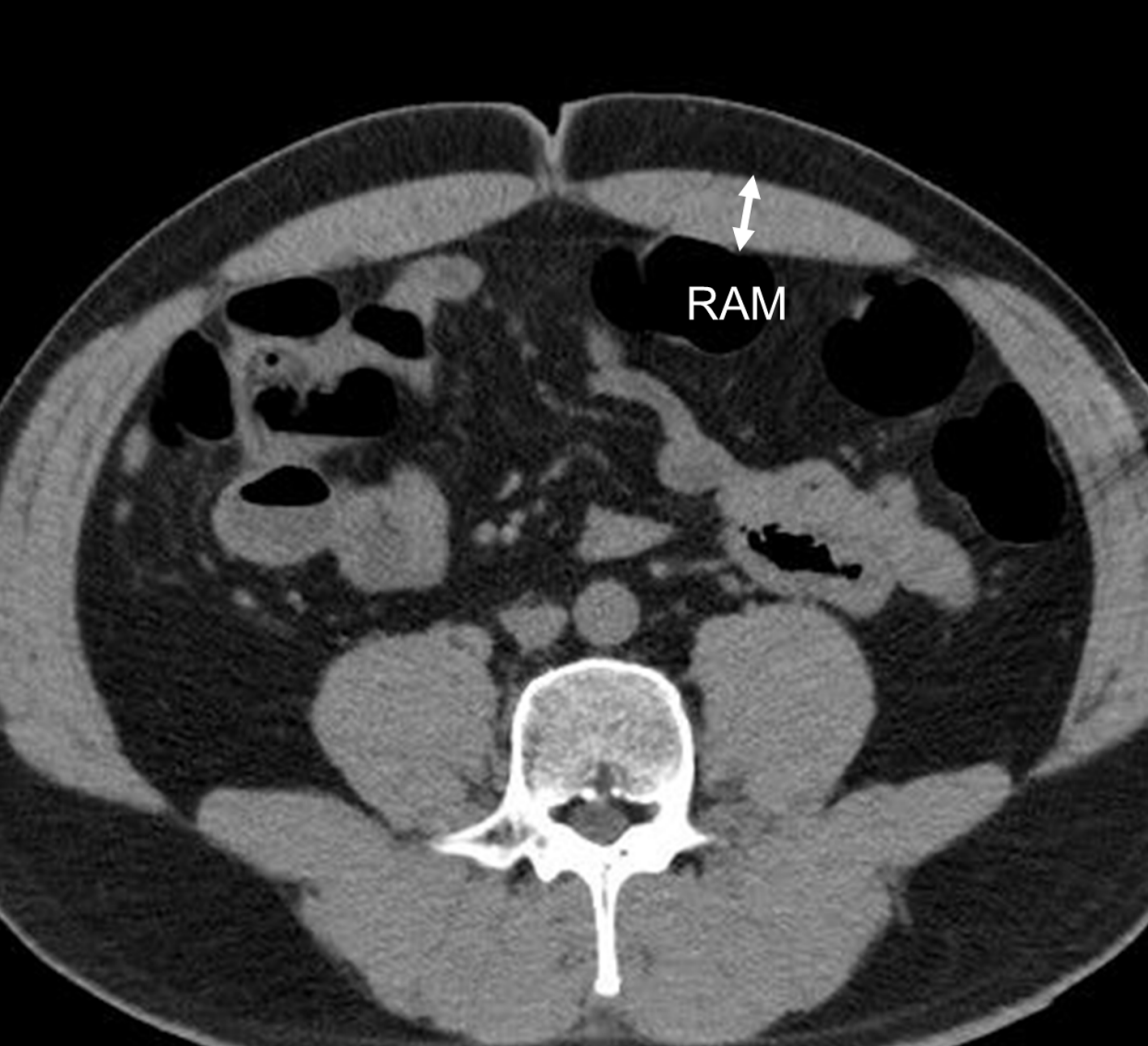
The thickness of the rectus abdominis muscle. The thickness of the rectus abdominis muscle were measured to the nearest 0.01mm from CT images with the picture archiving and communications system (PACS) viewer program (Marosis m-view 5.4). RAM, thickness of the thickest part of the rectus abdominis muscle thickness at the umbilicus level.

Rectus abdominis muscle thickness was measured to the nearest 0.01 mm from CT images using the caliper tool of the picture archiving and communications system (PACS) viewer program (Marosis m-view 5.4, Marotech, Seoul, Korea). At the umbilicus level, rectus abdominis muscle thickness at the thickest part was measured and defined as the RAM (Fig 1).

### Anthropometric evaluation

For anthropometric measurement, the body mass index (BMI) of each subject was calculated by dividing body weight by height squared (kg/ht^2^). Each subject was stable for more than 10 minutes, after which his blood pressure was measured with an automatic blood pressure monitor (FT-500R, Selvas Healthcare, Seoul, Korea) in the sitting position. Waist circumference was measured at the midpoint between the lowest rib and the iliac crest, while the subject was standing. Skeletal muscle mass was determined by bioelectrical impedance analysis (Inbody 720, Inbody, Seoul, Korea). The skeletal muscle index (SMI) was calculated as the ratio of appendicular skeletal muscle mass to total body weight [10].

### Laboratory measurements

Laboratory testing was performed on blood samples collected after 8h of fasting. Fasting glucose, creatinine, triglyceride, total cholesterol, HDL-cholesterol, aspartate aminotransferase (AST), alanine aminotransferase (ALT), and gamma-glutamyl transferase (GGT) levels were measured by enzymatic methods on an AU 5800 chemistry analyzer (Beckman Coulter, Brea, CA, USA). WBC count was measured by flow cytometry using fluorescent dyes (XN-9000, SYSMEX, Kobe, Japan).

### Statistical analysis

All variables are expressed as means± standard deviations (SDs) in a normal distribution or median with interquartile range (IQRs, 25^th^-75^th^ percentiles) in non-normal distribution. Variables such as glucose concentrations, triglyceride values, and RAM were log-transformed into normally distributed values. The baseline characteristics and RAM of the subjects with MS versus without MS were compared using the t-test. RAMs according to the number of component (0, 1–2, ≥3) of MS were analyzed by ANCOVA after age and BMI were adjusted. Partial correlation coefficients were calculated to evaluate the association between RAM and the components of MS or cardiometabolic variables after controlling for age and/or BMI. Statistical significance was defined at the 0.05 level of confidence. We performed a stepwise multiple logistic regression analysis to exclude any influence of potential confounding variables. Variables included in the diagnostic criteria for MS and AST that caused multicollinearity with ALT (r = 0.76, p<0.01) were excluded from the regression model Ⅰ. Variables such as age, BMI, SMI, total cholesterol, WBC, ALT, GGT and creatinine were included in the regression model Ⅰ. In the regression model Ⅱ, we included the components of MS and excluded waist circumference, diastolic blood pressure and AST that occurs multicollinearity with BMI(r = 0.85, p<0.01), systolic blood pressure(r = 0.83, p<0.01) and ALT(r = 0.70, p<0.05), respectively. Variables included in the regression model Ⅱ were age, BMI, SMI, total cholesterol, WBC, ALT, GGT, creatinine, systolic blood pressure, fasting glucose, HDL cholesterol, and triglyceride. All calculations were performed using the SAS 9.1 statistics package (SAS Institute, Inc., Cary, NC, US).

## Results

### Clinical characteristics

Table 1 presents the clinical characteristics of subjects with MS and subjects without MS. Age, total cholesterol, and creatinine did not show any significant differences between the two groups. BMI, WC, BP, fasting glucose, TG, WBC count, AST, ALT, and GGT were significantly higher in middle-aged men with MS than in those without MS (All *p*<0.01). In contrast, SMI and HDL-cholesterol were significantly lower in middle-aged men with MS than in those without MS (*p*<0.01).

**Table 1:** Clinical characteristics of subjects according to the presence of metabolic syndrome.

Characteristic	Without MS(N = 474)	With MS(N = 251)	*p*-Value
Age(years)	48.8±6.1	49.5±6.5	0.20
Body mass index(kg/m^2^)	24.0±2.4	26.9±2.5	<0.01
Waist circumference (cm)	85.0±6.2	93.2±5.9	<0.01
Skeletal muscle index (%)^a^	33.3±2.2	31.2±1.8	<0.01
Blood pressure(mmHg)			
Systolic	119.1±12.4	130.9±12.8	<0.01
Diastolic	72.1±9.0	79.4±9.5	<0.01
Fasting glucose(mg/dl)	98(93–105)	108(102–120)	<0.01
Total cholesterol(mg/dl)	195.1±34.9	200.1±41.7	0.10
HDLcholesterol(mg/dl)^b^	51.9±9.6	45.8±8.3	<0.01
Triglyceride(mg/dl)	108(80–142)	189(147–243)	<0.01
WBC count(10^3^/ul)^c^	5945.7±1439.6	6567.2±1546.5	<0.01
AST(IU/L)^d^	27.4±13.0	30.6±12.4	<0.01
ALT(IU/L)^e^	27.6±17.7	37.5±22.5	<0.01
GGT(IU/L)^f^	40.8±49.2	58.3±48.1	<0.01
Creatinine(mg/dL)	0.90±0.11	0.89±0.12	0.12

Data in the normal distribution are expressed as means ± standard deviations (SDs) and data not in the normal distribution as medians (IQRs, 25th– 75^th^percentiles). *p*-values were calculated using the *t*-test.

^a^ Skeletal muscle index(SMI) is the ratio of estimated appendicular skeletal muscle mass to total body weight, expressed as a percentage.

^b^ High density lipoprotein cholesterol.

^c^White blood cell.

^d^Aspartate aminotransferase.

^e^Alanine aminotransferase.

^f^Gamma-glutamyl transferase.

### Rectus abdominis muscle thickness

**Fig 2:**
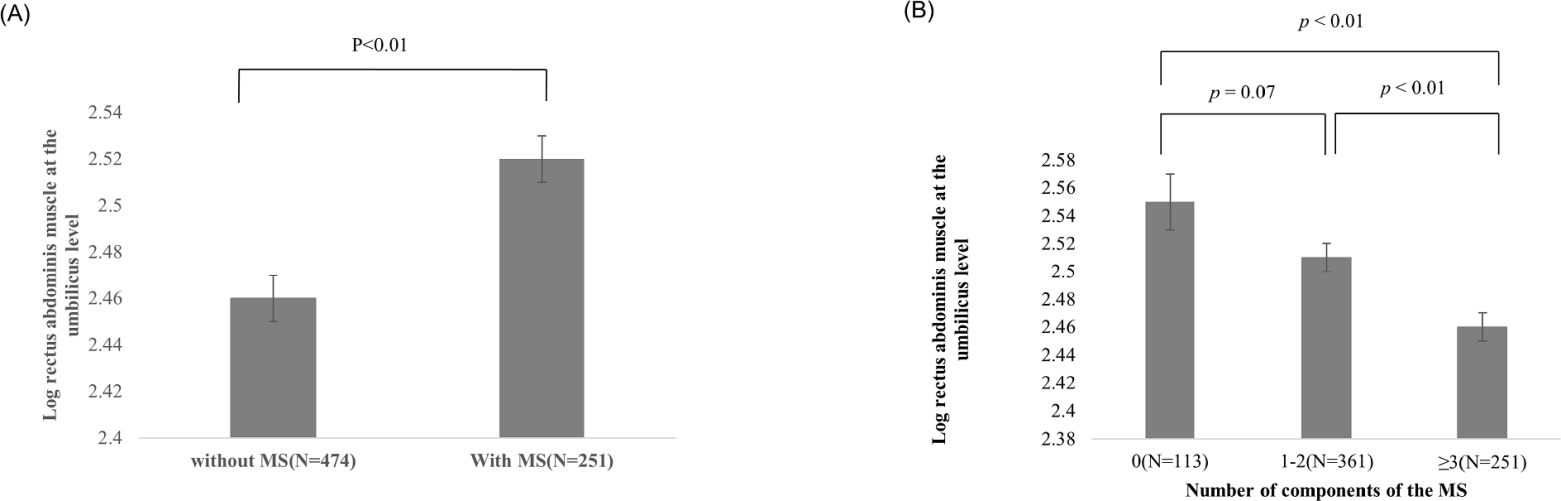
Rectus abdominis muscle (RAM) at the umbilicus level according to the presence of metabolic syndrome (MS) and according to the number of components of MS that were present. Values of RAM were analyzed after a log transformation. (A) RAM was significantly lower in subjects with MS (2.46 ± 0.01) than without MS (2.52 ± 0.01) (p <0.01). (B) As the number of MS components increased, RAM decreased *(P*-values for trending <0.01). *P*-values were calculated using ANCOVA after age and BMI were adjusted. The RAM of the group without any components of the MS diagnostic criteria (2.55 ± 0.02) was thicker than the RAM of the group which had three or more components of MS (2.46 ± 0.01) (*p*<0.01). The RAM of the group which had two or less of the components of MS (2.51 ± 0.01) was thicker than the group which had three or more components of MS (2.46 ± 0.01) (*p* <0.01).

In subjects with MS, RAM was thinner (2.46±0.01) than in those without MS (2.52±0.01) (*p*<0.01; Fig 2A). RAM was also compared according to the number of MS components that were present. RAM was found to decrease as the number of MS components increased (*p*-value for trend<0.01; Fig 2B). The RAM of the group without any components of the MS diagnostic criteria (2.55 ± 0.02) was thicker than RAM of the groups who had three or more components of MS (2.46 ± 0.01) (*p*<0.01; Fig 2B). Moreover, RAM was thicker in groups in which two or less of the MS criteria were satisfied (2.51 ± 0.01) compared to the group in which three or more were satisfied (*p* <0.01; Fig 2B).

### Relationships between rectus abdominis muscle and other parameters

Partial correlation analysis revealed that RAM was positively correlated with BMI(r = 0.21, *p*<0.01) after controlling for age. In addition, RAM showed positive correlations with SMI(r = 0.26, *p*<0.01) and creatinine (r = 0.12, *p*<0.01) after controlling for age and BMI. In contrast, after controlling for BMI, RAM was negatively correlated with age(r = -0.11, *p*<0.01). RAM also showed negative correlations with waist circumference(r = -0.22, *p*<0.01), fasting glucose (r = -0.10, *p*<0.01), and triglycerides(r = -0.15, *p*<0.01) after controlling for age and BMI (Table 2).

**Table 2:** Correlations between rectus abdominis muscle thickness and various parameters.

Variable	*r*	P-Value
Age(years)^a^	-0.11	<0.01
Body mass index (kg/m^2)^^b^	0.21	<0.01
Waist circumference (cm)^c^	-0.22	<0.01
Skeletal muscle index(%)^c^	0.26	<0.01
SBP(mmHg)^c^^,^^d^	-0.03	0.50
DBP(mmHg)^c^^,^^e^	-0.03	0.43
Fasting glucose(mg/dl)^c^^,^^f^	-0.10	<0.01
Total cholesterol(mg/dl)	0.13	0.24
HDL-cholesterol(mg/dl)^c^^,^^g^	0.06	0.10
Triglyceride(mg/dl)^c^^,^^f^	-0.15	<0.01
WBC count(10^3^/ul)^c^^,^^h^	-0.06	0.12
AST(IU/L)^c^^,^^i^	0.01	0.79
ALT(IU/L)^c^^,^^j^	-0.05	0.14
GGT(IU/L)^c^^,^^k^	-0.01	0.77
Creatinine(mg/dL)	0.12	<0.01

Coefficients(*r*) and *p*-values were calculated using the partial correlation method.

^a^Coefficients were calculated after controlling for BMI.

^b^Coefficients were calculated after controlling for age.

^c^Coefficients were calculated after controlling for age and BMI.

^d^ Systolic blood pressure(mmHg).

^e^ Diastolic blood pressure(mmHg).

^f^Values were analyzed after log transformation.

^g^ High density lipoprotein cholesterol.

^h^White blood cell.

^i^Aspartate aminotransferase.

^j^Alanine aminotransferase.

^k^Gamma-glutamyl transferase.

### Stepwise multiple regression analysis to identify independent relationships of RAM with clinical variables

RAM was an independent factor associated with MS in the stepwise multiple regression model Ⅰ that included age, SMI, BMI, total cholesterol, WBC, ALT, GGT, and creatinine (OR: 0.861, 95%CI, 0.779–0.951, *p*<0.01) (Table 3). RAM was also an independent factor associated with MS in stepwise multiple regression model Ⅱ that included age, SMI, BMI, WBC, ALT, creatinine, GGT, systolic BP, fasting glucose, total cholesterol, HDL cholesterol, and triglycerides (OR:0.860, 95%CI, 0.767–0.965, *p* = 0.01) (Table 3). Also, we found that SMI showed no independent association with MS, but RAM showed independent association in the in the stepwise regression model Ⅱ including the components of MS.

**Table 3:** Stepwise multiple logistic regression analysis for metabolic syndrome.

	ß	S.E.	p-Value	OR(95% C.I)
Model Ⅰ^a^: excluding components of metabolic syndrome				
RAM^c^	-0.15	0.05	<0.01	0.861(0.779–0.951)
Age	0.03	0.02	0.04	1.032(1.001–1.064)
SMI^d^	-0.16	0.06	0.01	0.851(0.752–0.963)
BMI	0.43	0.06	<0.01	1.549(1.385–1.733)
WBC^e^	0.22	0.06	<0.01	1.247(1.102–1.411)
GGT^f^	0.01	<0.01	<0.01	1.007(1.003–1.010)
Model Ⅱ^b^: including components of metabolic syndrome				
RAM^c^	-0.15	0.06	0.01	0.860(0.767–0.965)
Age	0.04	0.02	0.04	1.039(1.001–1.077)
BMI	0.43	0.05	<0.01	1.536(1.379–1.710)
SBP^g^	0.07	0.01	<0.01	1.076(1.055–1.098)
Fasting glucose	0.05	0.01	<0.01	1.048(1.031–1.065)
HDL cholesterol	-0.07	0.01	<0.01	0.932(0.905–0.960)
Triglyceride	0.01	<0.01	<0.01	1.012(1.008–1.015)

All variables left in the Model Ⅰ and Ⅱ were significant at the 0.15 level. No other variable met the 0.15 significance level for entry into the model Ⅰ and Ⅱ.

^a^Model Ⅰ excluded the components of MS and AST that occurs multicolinearity with ALT (r = 0.76, p<0.01).Variables included in the stepwise model Ⅰ: age, BMI, SMI, total cholesterol, WBC, ALT, GGT and creatinine.

^b^Model Ⅱ included the components of MS. Model Ⅱ excluded abdominal circumference, diastolic blood pressure and AST that occurs multicolinearity with BMI(r = 0.85, p<0.01), systolic blood pressure(r = 0.83, p<0.01) and ALT(r = 0.70, p<0.05), respectively. Variables included in the stepwise model Ⅱ: age, BMI, SMI, total cholesterol, WBC, ALT, GGT, creatinine, systolic blood pressure, fasting glucose, HDL cholesterol, and triglyceride.

^c^RAM was an independent variable.

^d^Skeletal muscle index(SMI), the ratio of estimated appendicular skeletal muscle mass to total body weight, expressed as a percentage.

^e^White blood cell.

^f^Gamma-glutamyl transferase.

^g^Systolic blood pressure.

## Discussion

In this study, we found that RAM measured using CT images was associated with MS in middle-aged men. We also found that SMI showed no independent association with MS, but RAM showed independent association in the statistical analysis performed including the components of MS. Our findings therefore suggest that the RAM measured using single abdominal CT image may be a predictor for the MS like SMI.

Recent studies reported the thickness or cross-sectional area of abdominal muscle measured from a single abdominal cross-sectional CT image. These studies demonstrated that these factors were related to health-related outcomes. Total muscle mass can be estimated from a single abdominal CT cross-sectional image at the L3 or L4 level [17]. A previous study reported that abdominal muscle measurements evaluated in this way were associated with mortality, postoperative complications, and hospitalization [18]. In addition, this method for assessing abdominal muscle is relatively objective and simple, enabling the prediction of frailty syndrome in elderly patients [18]. Another study demonstrated that the cross-sectional area of abdominal muscle is inversely correlated to type 2 DM independently of visceral fat in postmenopausal women [19]. Moreover, sit-up performance, which reflects rectus abdominis muscle strength, has been reported to predict MS in young adults [20]. These preliminary results suggest that abdominal muscle assessment can be used as an index for predicting MS. In a study of obese patients, they found out whether the thickness of specific muscles as measured by ultrasound was shown to be related to the MS risk score [14]. This study demonstrated that rectus abdominis muscle thickness only showed a negative correlation with the risk score of MS [14]. In particular, the study found that rectus abdominis muscle thickness better reflects the MS risk score than SMI [14], which is consistent with our findings. It is plausible that the decrease in skeletal muscle plays an important role in the pathogenesis of MS. The age-related, site-specific nature of muscle loss means that the degree of influence on MS may vary according to the muscle region. In particular, age-related, site-specific muscle loss has been reported to occur more frequently in the quadriceps and abdominal muscles, and also more frequently in anterior muscles than in posterior muscles [11, 12]. Previous studies have also shown that abdominal muscle exercise can have positive effects on MS or frailty syndrome [18, 19]. Resistance exercises such as sit-ups and leg raises, which build rectus abdominis muscle mass, can help improve MS by preventing or suppressing age-related muscle loss[21].

The mechanism underlying the relationship between MS and rectus abdominis muscle is not well understood and the current data do not enable the precise dissection of this relationship. However, studies on the relationship between loss of skeletal muscle and MS have suggested that testosterone may contribute to the development of MS in middle-aged men. This study was conducted on middle-aged men between the ages of 40 and 64, when the loss of skeletal muscle begins [22]. The main physiologic anabolic hormone, testosterone, is thought to be a main factor in the age-related loss of muscle mass in men. Studies have shown that the decline of testosterone levels in aging men is associated with muscle mass loss, increased insulin resistance, and the development of MS [23]. However, it is not clear whether these relationships are causal or the sequences in which these processes occur. In studies using testicular feminized mice, GLUT4 expression was decreased in muscle, and the addition of testosterone to cultured skeletal muscle cells increased GLUT4 expression and promoted glucose uptake [24]. In addition, testicular feminized mice exhibited significant decreases in key enzymes involved in glycolysis in muscle cells. When these rats were treated with testosterone, the activities of glycolysis-related key enzymes in cultured skeletal muscle cells were increased and their elevated blood glucose levels were decreased [25, 26]. Previous animal studies were performed using cultured muscle cells or muscles of the head, jaw, forearm, thigh, and leg regions. Further studies on the relationship between testosterone and insulin resistance in abdominal muscles are needed.

A strength of this study is that it is the first study to investigate the relationship between site-specific muscle loss and MS by analyzing rectus abdominis muscle thickness using CT image in middle-aged men. Abdominal muscles are known to be less affected by weight than thigh muscles [27]. Thus, evaluation of abdominal muscle is worthwhile when evaluating site-specific muscle loss.

This study also has limitations. First, because this study is a cross-sectional study, it was impossible to determine whether the relationship between MS and site-specific muscle loss is causal. Second, because all of the subjects in this study were Korean middle-aged men, our study may not be generalizable to other races, sexes, or age groups. Further prospective studies of the relationship between rectus abdominis muscle thickness and MS are required subjects of various races, ages, and sexes. Third, we calculated SMI using BIA instead of DXA, the gold standard of limb muscle mass measurement [10].Since the BIA equation for estimating appendicular muscle mass is derived from Caucasians [10], appendicular muscle mass may not have been accurately measured in this study group. In addition, we have not assessed the thickness of the other abdominal muscles and paraspinal muscles at the umbilicus level, and the visceral fat area, which are highly associated with MS. In order to increase the validity of our results, further studies will be needed to be performed including analysis of other site muscles and visceral fat at the umbilicus level which can be evaluated in a single abdominal CT image. Finally, muscle mass evaluation using CT images led to underestimation of intramuscular adipose tissue (IMAT), since only rectus abdominis muscle thickness was measured. However, previous studies have reported that thigh IMAT increases with increasing BMI [28, 29] and that IMAT at the L3 level (including rectus abdominis muscle) was not associated with BMI[30].

In conclusion, we found that middle-aged men with MS had thinner rectus abdominis muscles than those without MS. Furthermore, RAM was associated with the number of components of MS present in middle-aged men. Therefore, that the RAM may be a valuable for risk assessment of MS and exercise related improvement of abdominal muscle may be helpful prevention of MS development in middle-aged men.

## Supporting information

S1 File RAM database. (XLS)
